# Recent developments in the structural science of materials

**DOI:** 10.1107/S2052252515010891

**Published:** 2015-06-30

**Authors:** C. R. A. Catlow

**Affiliations:** a Department of Chemistry, University College London, 20 Gordon Street, London, WC1H 0AJ, UK

**Keywords:** materials, structural science

## Abstract

This Editorial surveys the current status and recent developments in the structural science of materials as exemplified by the articles recently published in **IUCrJ**.

The articles published recently in **IUCrJ** illustrate the rapid advances that are being made in this increasingly diverse field. There are very extensive and exciting developments in experimental technique, accompanied by progress in data processing and analysis and a growing integration with computational simulation. The field explores a wide range of classes of material including, of course nano-structured materials; while a multi-disciplinary approach – long a major feature of the structural materials science – is evident in all the recently published studies.

*Technical innovation* is central to the field. An important and highly topical example is the study of Dejoie *et al.* (2015 ▸) demonstrating the potential of ‘Serial Snapshot Crystallography in Materials Science’ This pioneering work used synchrotron radiation to simulate the type of brilliant ultra-fast X-ray pulses that will be available from X-ray free electron lasers (XFELs) which are being developed around the world. Using Laue techniques, which will be available on the SwissFEL source, they were able for several inorganic materials to collect data which could be at least partially analysed from a single shot of a 10–50 femtosecond pulse. As they comment, the success of this work offers ‘tantalizing possibilities for time-resolved studies’. And indeed with the increasing availability of XFEL sources in the coming years a new area of structural materials science will be opened up.

A perennial problem in the structural science of solids is the treatment of *disordered systems*, which embrace some of the most widely studied and significant materials, including ceramics (*e.g.* the very widely studied stabilized zirconia system) and zeolite catalysts. Analysis of diffuse scattering plays a key role here and the current state-of-the-art is reviewed by Welberry & Goossens (2014 ▸), who illustrate both the quality of the data which are available with current sources and instrumentation, an illustration of which is given in Fig. 1 ▸ for the case of benzil collected on the 11-ID-B beamline of the Advanced Photon Source (APS). In conjunction with modelling, using Monte-Carlo and *ab-initio* techniques, data of this quality will offer new possibilities in developing detailed models of disordered materials.

Technique development is also at the forefront of several other contributions, including the work of Rafaja *et al.* (2014 ▸) who exploited the high sensitivity of X-ray diffraction to macroscopic and microscopic lattice deformations in their study of metastable thin films; while Tria *et al.* (2015 ▸) report the development of ensemble modelling techniques in the analysis of X-ray solution scattering data from flexible macromolecules. Developments in data analysis and structure solution are highlighted in the feature article of Rius (2014 ▸) which surveys the current status of Patterson-function direct methods (PFDM); while more fundamental theoretical aspects are explored in the article of Marzouki *et al.* (2014 ▸) concerning the analysis of structural continuity in twinned crystals. Other technical advances are contributing to the field, a notable example being the growth of tomography, as illustrated in the recent developments in pair distribution function computed tomography of Jacques *et al.* (2013 ▸)

One of the most intriguing studies, which again has required extensive instrumental development, is provided by the work of Bish *et al.* (2014 ▸), who reported the first X-ray diffraction measurement on Mars. The article was based on data obtained from ‘CheMin’ – a miniaturized X-ray diffraction/fluorescence instrument which was on board the Curiosity Rover of the Mars Science Laboratory, which landed in Gale crater on Mars in 2012. The instrument analysed four samples and was able to achieve Rietveld and full pattern analysis of the diffraction data. A complex mineralogy was revealed and a notable feature was the identification in mudstone samples of phyllosilicates, suggesting alteration in liquid water.

As commented above, *nano-science* remains centre stage in our field. The article of Ringe (2014 ▸) reviews the challenges posed by controlling and characterizing the external shape and crystal structure of such small nanocrystals and highlights the key role of nucleation and growth processes in determining nano-crystal structures and properties. Kuzmin & Chaboy (2014 ▸) review the role of X-ray spectroscopy (both XANES and EXAFS) in analysing nano-particle structures; their work again emphasizes the role of modelling and simulation in assisting structural analysis. The role of XAFS in elucidating complex nano-particulate structures of importance in catalytic applications is also shown in recent work on alloy nano-particles of Gibson *et al.* (2015 ▸). Another theme within nano-science is illustrated by the work of Cao *et al.* (2014 ▸), who show how a combination of wide-angle X-ray diffraction (WAXD) and TEM, can reveal structural details of self- assembled nylon-12 rods in self-organized nanoporous alumina cylinders. TEM micrographs of these fascinating systems are shown in Fig. 2 ▸.

A wide *range of materials* are reported and discussed, including both inorganic materials and soft matter. A particularly fascinating class are the multi-ferroics investigated by Gilioli & Ehm (2014 ▸). These solids show two or more of the ferroic order parameters (ferroelectricity, ferromagnetism and ferroelasticity) simultaneously. The article reviews high pressure/high temperature synthetic methods and *in situ* high pressure/temperature studies of structure and properties. They show how the latter reveal new knowledge of the coupling and interaction between electric, magnetic and structural properties of these materials.

Overall, the recently published articles within our field show how the advances in sources, instrumentation and technique, coupled with developments in theory, data analysis and modelling are having a major impact on materials science, where structural information of the quality that is now available for complex systems is of key importance in understanding and predicting materials properties.

A full list of papers within the materials and computation theme of **IUCrJ** can be found at http://journals.iucr.org/m/services/articles_mater_comput.html. The journal welcomes more submissions in this rapidly expanding field.

## Figures and Tables

**Figure 1:**
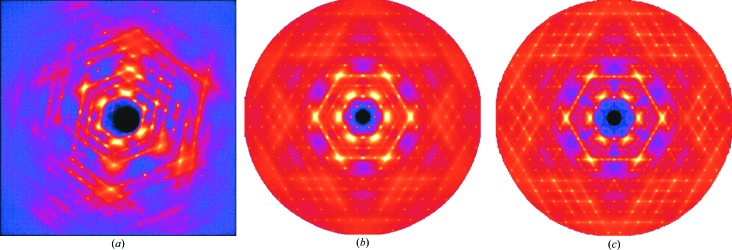
Diffraction patterns of benzil obtained from data recorded at the 11-ID-B beamline of the Advanced Photon Source (APS). (*a*) A single data frame recorded on a Perkin–Elmer amorphous silicon two-dimensional area detector using 58.26 keV X-rays (λ = 0.2127 Å). (*b*) The *hk*0 reciprocal section at 300 K, reconstructed from a data set comprising 740 such frames. (*c*) The same section recorded at 100 K. The maximum 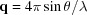 recorded was 8.52 Å^−1^. Note that the intensities in these images are displayed on a logarithmic scale. Reproduced from Welberry & Goossens (2014 ▸).

**Figure 2:**
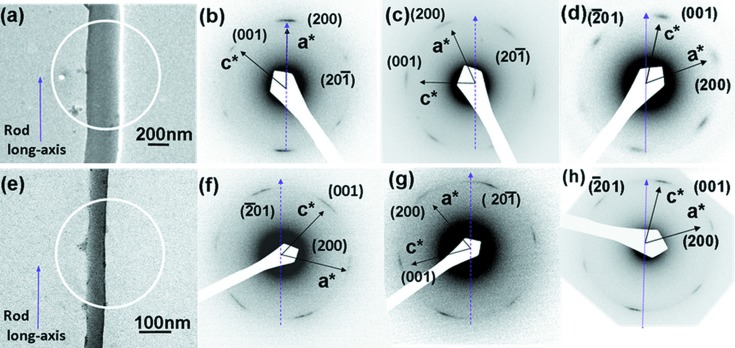
BF TEM micrograph (*a*) of a 300 nm rod and SAED patterns (*b, c, d*) with the [010] zone of γ-form crystals within the 300 nm rods. The crystal growth direction in the long axis of the rod is shown along (*b*) the [200] direction and in between (*c*) the [200] and [20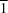] and (*d*) the [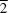
01] and [001] directions. BF TEM micrograph (*e*) of a 65 nm rod and SAED patterns (*f, g, h*) with the [010] zone of γ-form crystals within the 65 nm rod. The crystal growth direction is shown in between (*f*) the [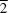
01] and [001], (*g*) the [200] and [20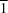] and (*h*) the [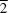
01] and [001] directions. Dashed lines denote the long axis of the rod. Reproduced from Cao *et al.* (2014 ▸)
